# Stigma, race, and testimonial injustice in mental health detention: Professionals experience of Compulsory Assessment and Treatment under The Mental Health Act 1983

**DOI:** 10.1371/journal.pmen.0000567

**Published:** 2026-03-30

**Authors:** Caroline Leah, Jeremy Dixon, Elaine Craig, Kim Heyes, Debbie Best, Anna Bergqvist, Alina Haines-Delmont

**Affiliations:** 1 Department of Social Care and Social Work, Manchester Metropolitan University, Manchester, United Kingdom,; 2 Centre for Adult and Social Care Research (CARE), Cardiff University, Cardiff, United Kingdom,; 3 School of Nursing and Public Health, Manchester Metropolitan University, Manchester, United Kingdom; 4 School of History, Politics and Philosophy, Manchester Metropolitan University, Manchester, United Kingdom; National Psychological Association of Ukraine, UKRAINE

## Abstract

This article examines how stigma, racism, and epistemic injustice shape health and social care professionals’ experiences of Compulsory Assessment and Treatment (CAT) within mental health and policing systems, with particular attention to the racialised experiences of Black men. Despite longstanding recognition of institutional racism and structural inequality, the persistent over‑detention of Black men under mental health legislation remains inadequately addressed. This study advances the field by shifting focus from procedural reform to epistemic critique, analysing how credibility, knowledge, and understanding are unevenly distributed, racialised, and denied within CAT practices. Drawing on a multi‑method Experience-Based Co‑Design (EBCD) study, the article reports qualitative findings from interviews with 13 mental health professionals, an EBCD feedback event, and a co‑design workshop involving 46 participants. Centring experiential knowledge, the analysis applies reflexive thematic methods informed by epistemic injustice, Critical Race Theory, stigma inequality, and the Silences Framework. Three themes are identified: (i) Racial Stereotyping and Risk Assessment, (ii) Stigma and the Denial of Care, and (iii) Race Anxiety and Colour-blind Racism. These findings illustrate how racialised framings within CAT generate both testimonial and hermeneutical injustice, silencing Black men’s voices and rendering their experiences unintelligible within dominant professional paradigms. The analysis makes a significant scholarly and practical contribution by demonstrating that stigma functions as a structural and institutional logic shaping professional decision‑making. Meaningful change therefore requires systemic transformation, rather than a narrow focus on individual professional attitudes. The article calls for greater epistemic openness, urging professionals to recognise the limits of dominant frameworks and to incorporate lived experience and community‑based knowledge into practice. By reframing mental health and policing practices through an epistemic lens, the study identifies a transformative route toward more equitable, accountable, and just systems. Although situated in the UK, the insights have broader relevance for global mental health systems confronting racialised exclusion and structural inequity.

## Introduction

This article examines how Black men experience compulsory assessment and treatment (CAT) under the Mental Health Act 1983 (MHA), with a particular focus on how racism, trauma, and epistemic injustice shape their access, encounters, and outcomes within mental health systems. The study seeks to explain why longstanding disparities persist, how institutional practices reinforce harm, and what changes are needed to produce more equitable and culturally grounded Mental Health Act response.

Substantial literature documents the disproportionate mental health inequities faced by Black men subjected to CAT across the UK, Europe, and North America [1–3]. These inequities include more adverse and coercive pathways to care [4], limited access to psychological therapies [5], fear and mistrust of mental health services [6], and the internalisation of racialised expectations to appear resilient and suppress vulnerability [7]. The frequent involvement of police during CAT further compounds trauma responses and reinforces harmful narratives of Black men as dangerous [8].

Black men also experience both institutional and interpersonal racism, recognised in the literature as forms of trauma [9], and persistent stigma, mistrust, and risk‑averse practices [10]. Cultural competence, although intended to improve cross‑cultural care, remains dominated by Eurocentric norms that function as a form of cultural capital, marginalising Black cultures and knowledge systems [11].

These patterns are mirrored internationally. In North America, Black men have historically been framed through tropes of aggression, hypersexuality, and violence—racialised narratives that have long justified surveillance, coercion, and social control [12]. The disproportionate killing of unarmed Black men by police in the United States [13] underscores the global resonance of these racialised logics and their psychological impacts.

Within the UK, these dynamics are rooted within a broader historical and structural context shaped by colonialism and post‑war migration from former British colonies. The institutional racism embedded in public systems continues to generate discrimination and exclusion, with anti‑Black racism exerting profound effects on the mental health, safety, and identities of Black men [14,15]. This historical grounding is essential for understanding how epistemic injustice operates within CAT and how anti‑Black racism is harmful to the mental health of Black men across international settings.

The Mental Health Act 1983 governs involuntary assessment, treatment, and safeguarding in England and Wales, allowing detention when individuals are deemed to pose risks to themselves or others, or lack capacity to make informed decisions. Despite repeated policy attention, Black men remain 3.5 times more likely to be detained under the Act compared with White men [15], a disparity that has remained remarkably stable across decades [16]. Understanding the structural and epistemic forces that sustain this enduring inequality provides the rationale for the approach adopted in this study.

The sections that follow outline the study’s conceptual framework, positionality and terminology, methodology, findings, recommendations, and conclusion.

### Conceptual framework

#### Epistemic injustice.

Epistemic injustice [17] refers to the ways individuals are wronged in their capacity as knowers, silencing their voices in both clinical and research settings. In the context of CAT this concept illuminates how Black men’s expressions of distress, personal narratives, and accounts of racialised experience are often dismissed, discredited or misinterpreted [18]. Fricker [19] elaborated on the harm that this can do and identified that immediate harm can be experienced in not being believed and thus being discredited. Consequently, on a deeper level “to be wronged as a knower is to be wronged in a capacity essential to human value” (p. 44); that is, to be denied opportunities not only to communicate, but to be heard. In the context of CAT this concept illuminates how Black men’s expressions of distress, personal narratives, and accounts of racialised experience are often dismissed, discredited or misinterpreted [18]. As Fricker states, “testimonial injustice occurs when prejudice causes a hearer to give a deflated level of credibility to a speaker's word” [17p.1]. In the psychiatric context, this form of injustice is often explained in terms of “the stigma of mental illness” to be resolved within individual encounters and through anti-stigma campaigns and awareness raising, although psychiatric diagnosing itself can be a principal enabler of testimonial injustice, especially for multiply oppressed groups like Black men.

Hermeneutic injustice arises when collective interpretive resources, such as mental health assessment tools, fail to capture the lived experiences of individuals who are subject to CAT, making it hard for them to make sense of their own suffering. Russo [20] argues that psychiatric diagnoses disproportionately target Black individuals, reinforcing systemic injustice and opportunities not only to communicate and be heard but also, to put a voice to one’s own experience and enter into dialogue, in ways that might increase an understanding of one’s experience. The dominance of academic and clinical voices over lived experience narratives further contributes to the silencing of the Black male experience in the global discourse [21].

Building on epistemic injustice, our study situates this theory within a broader constellation of intersecting structural and conceptual forces that shape the mental health experiences of Black men. These concepts are of vital relevance to the work of mental health professionals as many of the vulnerable populations we work with are subject to forms of injustice; and these injustices can be outside of our awareness. Our conceptual synthesis therefore draws on the following interrelated domains:

#### Stigma inequality.

Stigma is a well-documented global barrier to help-seeking behaviour and engagement in care [22]. Stigma manifestations influence not only access to health and social justice but co-occur with additional intersecting stigmas, such as those related to gender identity, sexual orientation and poverty [23]. Stigma is understood not only as an interpersonal issue, as posited by Goffman [24], but as a systemic form of oppression that dehumanises and disempowers. Since some stigma scholarship [25] adopts a siloed approach—focusing on individual attitudes while overlooking the structural and political conditions in which stigma is produced—we draw on Tyler’s concept of stigma inequality [26] to reposition stigma as a mechanism of mental health injustice. Tyler [26] argues that stigma is not merely an interpersonal bias but a state‑mediated form of inequality that organises institutional responses and legitimises unequal treatment. We apply stigma as a form of mental health inequality [26] to amplify stigma as a structuring feature during CAT, shaping how professionals interpret and respond to Black men’s distress.

#### Critical race theory and structural racism.

Critical race theory [27,28] and the concept of structural racism [9,29] further serve to frame the mental health detention of Black men not merely as a product of individual prejudice, but as embedded within longstanding, institutionally encoded systems of oppression. This theory allow us to interpret mental health legislation and professional practice as racialised structures that normalise Black men’s inequality under the guise of professional neutrality.

Since Critical Race Theory (CRT) recognises racism as a systemic and enduring feature of society, embedded in law, policy, and institutional practice [28], it foregrounds the experiential knowledge of minoritised communities, challenges dominant ideologies of objectivity and neutrality, and advocates for social justice (through praxis). Whilst, structural racism, as defined by Bailey et al. [29], refers to the normalisation and legitimisation of historical, cultural, institutional, and interpersonal dynamics that advantage White people while producing cumulative and chronic adverse outcomes for people of colour. This concept includes applying ‘Whiteness as Normativity’ [30,31], where Whiteness functions as the unmarked standard against which all others are judged, reinforcing White hegemony in mental health practice.

Additionally, the linked concept of race anxiety [32,33] is important for surfacing professionals’ discomfort and fear during inter-racial encounters, where such anxieties can lead to risk-averse, biased decision-making. Building on Chantler’s [32] work on the structural dynamics that shape therapeutic encounters, race anxiety describes the discomfort, hyper‑vigilance, or defensiveness that professionals may experience when issues of race, racism, or racial inequality arise in assessment contexts. For some professionals, this may take the form of anxiety about “saying the wrong thing,” being perceived as racist, or engaging directly with racialised power dynamics. Such anxieties can lead to avoidance, minimisation of racialised experiences, or premature shifts back to individualised clinical framings, thereby constraining the meaningful exploration of service users’ accounts of racism and its psychological effects. In this sense, race anxiety is not only a response to lived experiences of racism among racially minoritised groups but also a relational dynamic that shapes how professionals interpret distress, assess risk, and construct narratives of pathology. Attending to race anxiety is therefore essential to understanding how epistemic injustice may be reproduced within CAT, even when professionals intend to provide equitable and culturally responsive care.

#### Silences framework.

Finally, the Silences Framework [34] was applied across the whole research study to interrogate how dominant professional narratives suppress, distort, or render inaudible the experiences of marginalised groups, producing what Serrant describes as “screaming silences” within research, policy, and practice. When used alongside Tyler’s concept of stigma inequality, the Silences Framework helps expose how Black men’s accounts of trauma, fear, and mistrust are routinely sidelined or reinterpreted through risk‑based, racialised professional logics. Together, these concepts offer a multi-layered critique of how Black men are epistemically marginalised within mental health systems. They move beyond individual-level explanations to reveal the structural, racialised, and normative forces that shape clinical encounters, legislative frameworks, and knowledge production practices, thereby illuminating how inequality is continually reproduced through CAT responses.

This novel epistemic framing substantially advances existing literature beyond previous race related studies, via its shifting focus from individual level bias to the systemic devaluation of Black’s men's experiential knowledge and lived experiences of stigma and trauma. While Critical Race Theory highlights structural racism and the social construction of race, this article uniquely extends that analysis into the domains of applied mental health detention practice, to expose how testimonial and hermeneutical injustices operate during CAT – a terrain rarely mapped in race-health research [6,9].

#### Positionality and terminology.

In this study, we use the term ‘Black men’ to describe participants’ racial identity. This term encompasses men of Black African, Caribbean, or other Black or mixed heritage backgrounds living in the UK. In alignment with the Black Mental Health Manifesto [35], we capitalise ‘Black’ to reflect its use as a political identity—one that recognises the shared experiences of anti-Black racism and the enduring impact of colonialism.

In doing so, we acknowledge that intersectionality is both nuanced and individualised. Drawing on Crenshaw’s [27] foundational work, we recognise that co-existing elements such as class, gender, age, sexuality, ethnicity, and racialised identity intersect to shape how an individual’s mental health is perceived, assessed, and treated [36]. As Hall [37] reminds us, “race is indeed a sociohistorical concept, not a transhistorical discourse grounded in biology... [yet] the biological trace still functions even when silent, but now, not as truth, but as a guarantor of the truth.” (p.359). We therefore recognise the inherent epistemological tension of viewing racial categories as normative, given they are epistemically suspect in the sense they are based on demonstrably false ideas about essential human difference.

The aim of the article is to (i) examine how racialised framings during compulsory admission and treatment (CAT) contribute to epistemic injustice within mental health systems, particularly in the application of the Mental Health Act 1983, (ii) to explore the experiences and interpretations of mental health professionals (including AMHPs, psychiatrists, police officers, and a psychologist) regarding Black men in mental health crisis and to identify and analyse the epistemic dimensions of racial injustice, (iii) focus on how knowledge, credibility, and understanding are distributed or denied in mental health detention processes.

## Method

### Ethics statement

The study was approved by Health Research Authority and the Health and Care Research Wales (IRAS ID 310503, REC Reference: 22/HRA/2684). All participation was subject to informed written consent. Participant information packs were given to all participants identifying risks and benefits of participation, the consent process, how distress would be managed, and the secure data storage procedures. Individual written consent was gained for the professionals prior to the interviews. Their data was pseudo-anonymised, and each participant was given a unique identifier (e.g., Professional Interview, AMHP). Informed consent for EBCD event participants was obtained twice: written at the point of registration and verbal at the start of the event. Their views were pseudo-anonymised with group identifiers (e.g., Professional participant at the co-deign event). Attendees who did not want their views captured wore rainbow lanyards to indicate this.

The research team and participants had access to a counsellor as part of our distress protocol. Ethically, we critically reflected on how our own racial identities, privileges, and social locations shaped the research process. We were committed to engaging in culturally sensitive and respectful communication throughout, recognising the importance of trust, relational accountability, and the ethical imperative to honour participants’ lived experiences.

In this article we focus on one strand of the ImproveAct project, that interrogates the findings obtained from 13 semi-structured interviews with a variety of multi-disciplinary mental health professionals; including psychiatrists, approved mental health professionals (AMHPs), police officers and a psychologist involved in the detention process, 1 feedback/validation event with 5 professionals and a joint co-design event with 46 participants. This part of the study investigated professionals’ experiences of detaining Black men under the Mental Health Act, including their experiences and views of the impact of racial inequality before, during and after the detention process and their recommendations for improving care and treatment outcomes, experiences, and practice.

The study adopted an Experience-Based Co-Design (EBCD) approach, aligned with our aim to centre the experiential knowledge of those directly involved in or affected by mental health detention. Experience-Based Co-Design (EBCD) is a participatory research and improvement methodology used primarily in health and social care settings. It brings together service users, carers, and professionals to collaboratively design better services. The co‑applicant with lived experience was integrated throughout the analytic process as a core member of the research team. They participated in scheduled team meetings where coding decisions, emerging interpretations, and thematic development were discussed collaboratively. Their contributions formed a structured element of the analytic workflow, ensuring that lived experience meaningfully informed the refinement and validation of themes. Discussions relating to analytic decisions, positionality, and interpretive tensions were documented throughout.

EBCD is well-suited to interrogating and improving complex care systems, particularly where marginalised communities encounter structural inequality [38]. Our use of EBCD is situated within a critical epistemological framework, combining elements of critical race theory, Whiteness, race anxiety, and stigma inequality, which recognise that knowledge is socially situated and shaped by power [17,26,27,31,32]. We applied the Silences Framework [34,39,40] to guide the design, analysis, and reflexive engagement with ‘unsaid’, marginalised or suppressed narratives, including co-applicants with lived experience of detention. This approach was particularly important given the racialised dynamics of silence and erasure in the context of Black men’s mental health detention. The framework supported both methodological sensitivity and epistemic justice, encouraging the research team to surface latent meanings and acknowledge the sociopolitical contexts in which knowledge is produced, shared, or withheld.

### Setting, participants & data collection

Participants were recruited from a large NHS Mental Health Trust and a regional police service in England, where Black individuals comprise approximately 12% of the local population, which is three times the national average [15]. We employed purposive sampling to ensure the inclusion of professionals directly involved in the application of the Mental Health Act (MHA), particularly those with frontline experience of assessing and detaining individuals under the Act. A leaflet was sent to the heads of a mental health trust and a police force. Information on the study's aims, the funder, the methods, and the anticipated benefits and potential risks was included. Informed and ongoing consent was obtained prior to, during, and after the interview, due to the topic's sensitive nature. Participants were informed of their voluntary participation and the right to withdraw. No participants withdrew from the study. Financial renumeration was not offered.

There were 3 EBCD stages involved:

Firstly, thirteen professionals participated in one-to-one online interviews, as follows: five Approved Mental Health Professionals (AMHPs), three consultant psychiatrists, four police officers, and one clinical psychologist. All had direct experience of detention under the MHA, conducting or supporting CAT. The semi-structured, topic-based interviews were held over Microsoft Teams, for approximately one hour each between February and October 2023. Key areas explored included: professional roles in MHA detention, perceptions of racial inequality, understanding of risk and stigma, and recommendations for improving mental health detention experiences and outcomes for Black men. All interviews were recorded and transcribed verbatim by a third-party service.

Secondly, in December 2023, we held a feedback validation event with five professionals and five researchers to review and refine the emerging findings. We explored racial trauma, the complexities of mental health act assessments, and the perceived historical, cultural, institutional, and interpersonal aspects of Black men’s lived experiences.

Thirdly, in February 2024, a large-scale co-design event was held with 46 participants including Black men, family members, professionals involved in the detention process, community leaders, and third-sector organisations. To reduce the reproduction of institutional hierarchies and promote authentic participation, participants were not asked to declare their ‘role’ or status. Facilitation techniques focused on shared power, mutual respect, and the legitimisation of experiential knowledge. The co-design event was instrumental in developing meaningful engagement, building trust, and addressing the role of racism in CAT. It provided space for collective approaches and solutions to CAT and more broadly, improvements in mental health services. Through bringing together all stakeholders (including those involved in the study, as research participants) individual findings from the interviews, emerging themes and recommendations were sense checked to enable a ‘collective voice.’

### Data analysis

Data included interview transcripts and reflective field and feedback notes from the feedback and co-design events. We conducted reflexive thematic analysis [41], supported by NVivo. Two members of the research team (CL and JD) independently coded an initial set of five transcripts, generating 122 codes (110 overlapping). Through iterative meetings, a collaborative coding framework was developed and applied to the full dataset. Our analytical approach was contextualist, situated within a critical realist epistemology [42], which recognises that meaning is both socially produced and individually experienced. In taking this approach, we posit that individuals make meaning from their experience and are influenced by their social context but retain ‘agency’ to define their own realities. Thematic development was informed by (i) the Silences Framework [34], which prompted attention to omissions, tensions, and silenced narratives; (ii) Tyler’s [26] theory of stigma power as a machinery of inequality; and (iii) Fricker’s [17] concept of epistemic injustice, enabling us to theorise the ways in which Black men’s accounts were systematically interpreted by professionals. We held ongoing analysis meetings to surface differences in interpretation, engage in collective reflexivity, and ensure attention to positionality, particularly where research team members held insider or outsider status in relation to race, ethnicity, gender, profession, or lived experience. Final themes from the stage one interviews were reviewed and refined in collaboration with the broader ImproveAct project team (AH, AB, DB, LC) and at stage two during feedback validation, before presenting the findings at stage 3 to finalise and consider recommendations for improvement.

### Rigour and reflexivity

Reflexivity was achieved through participant quotations that captured professionals’ experiences. We assessed qualitative rigour using criteria adapted from Guba and Lincoln [43] and recent updates for participatory and anti-racist research. Credibility was established through member checking at feedback and co-design events; transferability was supported through detailed contextual descriptions; dependability through systematic documentation of analytic decisions; and confirmability through collaborative researcher analysis with lived experience interpretation, and reflexive journaling.

We acknowledge our own positionalities as researchers, some of whom are White, professional, or institutionally embedded, and others racialised and/or with lived experience. Our approach to reflexivity involved active participation in a workshop on anti-racism, structured discussion of power, privilege, and standpoint throughout the whole research process, supported by the Silences Framework, and coaching by Professor Laura Serrant on the interpretative aspects of the framework [34].

### Findings

Three themes are presented from the study with professionals, as follows:

1. Racial Stereotyping and Risk Assessment
2. Stigma and the Denial of Care
3. Race Anxiety and Colour-blind Racism

1 **Racial Stereotyping and Risk Assessment**

Professionals consistently described how Black men were framed as inherently risky, not necessarily due to their behaviour, but due to racialised assumptions embedded in institutional judgments. These assumptions triggered escalated responses, particularly from police officers. In the excerpt below, one AMHP recalled a case in which a routine MHA assessment was rapidly mobilised, without her consent, based on police perceptions of risk:

“*He started shouting saying ‘ No, I’m not coming to hospital’ At that point, two police officers burst in and tried to restrain him, even though he didn't need restraining...but they came in to restrain him, at which point he started to retaliate because there's two men on him and then I heard one of the police on the radio saying, ‘we need back up there's a big Black man resisting’...At the end of it 15 officers turned up, the police had blocked three roads, with vans, undercover and police cars. He was brought out by about six police officers all holding him, it’s so undignified... I think it’s institutional racism*” *(Professional’s interview 3, AMHP).*

This escalation illustrates how racialised notions of dangerousness activate disproportionate force, an example of structural racism operating through institutional practices of risk management. Risk here is not a neutral judgement but is based on racialised construct that triggers a coercive response [10]. These judgements are normalised within both policing and mental health services, where the stereotype of the ‘big, Black and dangerous’ man is perceived to justify restrictive intervention [6]. The same participant expanded on this dynamic:

*“You just think, if that was a big White man and they had said, we need back up, there’s a big White man here, would 15 coppers show up? I don't think so. I think it’s just institutional racism, that term ‘big Black man” (Professional’s interview 3, AMHP).*

We frame these narratives of the ‘big, Black, dangerous’ figure of speech as a racialised script produced through structural conditions that classify Blackness as pathological and threatening [6]. These examples demonstrate how structural racism is enacted through the racialised embodiment of risk, with Black men positioned as needing control rather than care. As CRT theorists note, when whiteness functions as an unmarked norm, Blackness becomes hyper-visible, pathologised, and policed [28].

One participant reflected on her experiences of receiving information as part of her risk assessment, highlighting how racial stereotyping is often unconscious, through the expression ‘there's no malice in it’:

*“I definitely see that warning language in referral when we’re talking about Black men, more so than I do in referrals when talking about White men. I don’t think it’s something people are conscious of, I think it just happens, which is awful but there’s no malice in it” (Professionals interview 8, AMHP).*

Although AMHP participants advocated for equity, it was acknowledged that subjective experiences, cultural frameworks, and institutional norms subtly influence CAT judgments. Conscious bias may emerge through overt beliefs or assumptions, whereas unconscious bias operates beneath awareness, shaping perceptions and responses in ways that reinforce systemic inequalities. Self-reflection within risk assessment processes reveals the critical role of both conscious and unconscious bias in professional decision-making. As one participant stated below:

*“Nobody wants to detain somebody based on any kind of bias, but some people are not aware they have these biases, and I’m not saying that I’m uniquely insulated. I undoubtedly have unconscious biases as well” (Professionals interview 10, Psychiatrist).*

Reflecting on stereotypical assumptions, in the quotation below, supports professionals to interrogate stereotypical biases, as part of CAT risk assessment, fostering a greater understanding of Black men’s mental health crisis:

*“Well I think there’s sort of multiple layers...I think there’s a perception of black men generally in society but also in mental health services as well, that they’re generally more aggressive, they’re described as aggressive more than not, and I think a willingness to understand...what’s driving that behaviour is clouded by that view” (Professionals interview 6, AMHP).*

This observation resonates strongly with critical race theory [27,28], which encourages the interrogation of taken-for-granted racialised assumptions. Such scrutiny is essential for improving culturally appropriate and anti-discriminatory practice (CAT), as it highlights how racialised stereotypes can distort clinical interpretations and shape decision-making.

Further examples presented in this theme, reveal how racialised assumptions about physicality, aggression, and risk are routinely embedded in professional discourse and practice. Statements such as:

*“They do seem very strong, very vocal, and a lot of time not willing to engage on even simple instructions” (Professional’s interview 4, Police participant).*

And:

*“They’re muscular, they go in the gym all the time, look at Linford Christie” (Professionals interview 13, Police participant).*

Reflect how racial profiling and stereotyping are normalised under the guise of professional objectivity. Such characterisations reflect racialised assumptions about individual behaviour and compliance, which are further compounded by the largely unconscious stereotypes that associate Black men with criminality, leading to biased judgments and stigmatising responses [44]:

*“A lot of the white male offenders we used to get in custody, they’re skinny little things and they're easier to restrain. I think Black men are more powerful, if you look at all the athletes and everything, I don't know, I feel like I’m verging on...”(Professional’s interview 4, Police participant).*

This narrative reveals how professional bias contribute to discriminatory treatment, reinforcing systemic racism under the guise of neutrality and professional objectivity.

Importantly, these racial stereotypes are not only confined to policing. They permeate other institutional spaces, including healthcare. In one example, an Accident and Emergency Department nurse, racially profiled a Black male patient presenting with suicidal ideation, stating:

*“He’s got his shirt off, he’s really muscular, he’s really threatening. Just be careful, he’s handcuffed at the moment” (Professional’s interview 3, AMHP).*

However,

*“When the AMHP went into a room to conduct the MHA assessment; ‘He said as long as the police are not in here, he’ll be fine. They uncuffed him and the first thing he did was got the blanket and put it over his chest and he looked scared, and he was unwell” (Professional’s interview 3, AMHP).*

This contrast reveals how racialised perceptions of threat obscure genuine distress and reinforces harmful stereotypes. The persistence of racial stereotyping reflects a broader cultural logic in which racialised difference is pathologised, and whiteness remains protected as a form of social property [44,45]. As one psychologist noted:

*“…there’s a perception of Black men generally in society but also in mental health services as well, that they are generally more aggressive, they’re described as aggressive more than not, and I think a willingness to understand where that behaviour comes from and what’s driving that behaviour is clouded by that view” (Professional interview 8, Psychologist).*

The normalisation of these dynamics across institutions reproduces patterns of healthcare that privilege white individuals while generating chronic, adverse outcomes for Black men experiencing mental health crises [29]. These hostile environments cultivate fear, injustice, and neglect, making it difficult for Black men to feel safe enough to seek care or treatment support.

This constitutes a form of testimonial injustice, as the credibility of Black men’s expressions of distress is systematically downgraded due to racialised assumptions rather than the content of their testimony [17]. Crucially, these assumptions are shaped by racial stereotyping, particularly the persistent framing of Black men as inherently more dangerous or high‑risk. Such stereotypes prime practitioners to interpret their behaviour through a risk lens, inflating perceptions of threat and diminishing their capacity to be seen as legitimate knowers of their own experiences. In this way, racialised constructions of risk directly produce the credibility deficit at the heart of testimonial injustice.

2 **Stigma and the Denial of Care**

Participants reflected on how stigma inequality is not only confined to interpersonal interactions but is reproduced and distributed across social and health systems, shaping how Black men are perceived, prioritised, and responded to in mental health care. Drawing on Tyler’s [26] concept of stigma power, the emotion of fear—particularly when embodied by Black men—is a key mechanism through which stigma operates.

Fear is both a response to past experiences of coercion and racialised surveillance. It is well known barrier to help-seeking [6]. Participants recounted numerous times significantly more coercive interventions from police officers towards Black men, where police involvement was perceived to generate stigmatisation:

*“That experience of being detained, certainly where police are involved, then that’s definitively likely that people are going to view the process as being more stigmatising, definitely” (Professionals interview 8, Psychologist).*

The stigma associated with CAT perpetuates a cycle of shame, avoidance, and mistrust, which scholars have shown delays Black men from accessing preventative support [6]. In this way, stigma becomes a structural force, reinforcing inequality by shaping both institutional responses and individual behaviours.

Our participants reflected on how stigma, as an institutional mechanism influences service responses, delays in care, and risk prioritisation—often with devastating consequences. One participant, for example, described how a Black man was denied care in an emergency department, not because of clinical need, but due to racialised assumptions and institutional bias:

*“The balance of risk was all about what he might do to them or staff versus the risk to him...and what ended up happening is he’d actually gone home, and he lived on his own and he took an overdose. Luckily...he didn't die, and he did get treatment eventually, but he was left for a quite a long time before that happened” (Professional’s interview 6, AMHP).*

This quote reveals a troubling shift in focus from the patient’s welfare to the perceived threat he posed to staff. This reflects Tyler’s [26] description of stigma power: the ability of stigma to shape institutional responses to justify neglect or coercion. It shows how stigma operates not only interpersonally but structurally, reproducing inequalities through routine practices. We found the embodied nature of stigma power meant that Black men’s distress is often interpreted through a lens of threat, by professionals, rather than vulnerability. The result is a mental health system that not only fails to support Black men but also reproduces the very conditions that make support inaccessible.

Some participants in our study, recognised the act of CAT itself can perpetuate violence and aggression, particularly for Black men who may experience it as a form of victimisation:

*“When they’re unwell and they don’t think they need admission, but the police turn up and they think, last time, this is what the police did to me. I need to run, I need to get out of here, it’s going to increase their fight or flight” (Professional’s interview 3, AMHP).*

The participant highlighted that a Black male patient’s aggression during detention may reflect self‑protection rather than hostility—a response to fear of restraint or further harm. This challenges dominant narratives of risk and shows how institutional interpretations of behaviour can be deeply racialised and stigmatising. It reveals how stigma shapes not only treatment but also how Black men’s actions are read, often in ways that reinforce harm and inequality.

For Black men, such responses can be understood as the externalisation of race‑based anxiety during Mental Health Act detention, where embodied fear triggers a fight‑or‑flight reaction [32]. The anxiety experienced by both the assessed individual and professionals can create a self‑fulfilling prophecy in which negative interactions confirm pre‑existing fears and contribute to care avoidance. These barriers are structurally embedded rather than the outcome of individual bias alone. Across triage systems and eligibility criteria, stigma shapes how risk is constructed, how urgency is determined, and how resources are distributed. Participants described how racialised trauma responses are frequently misinterpreted or pathologised. As one AMHP recalled, a man refused police entry because of previous negative experiences:

*“I’ve had bad experiences with the police; I don’t want the police in there”* (*Professional’s interview 3, AMHP).*

Yet within CAT, such protective, trauma‑informed behaviours are often reframed as indicators of risk, non‑compliance, or volatility. By recasting legitimate fear as problematic behaviour, stigma becomes structurally encoded, shaping how Black men’s actions are perceived and responded to. This dynamic reflects Tyler’s [26] concept of stigma power; whereby institutional actors draw on stigmatising narratives that position Black men as inherently threatening or uncooperative. Instead of recognising trauma, professional contexts absorb these responses into a broader racialised risk discourse, obscuring vulnerability and reinforcing unequal treatment.

Participants also commented on coercive police involvement, noting:

*“The biggest fear for Black men must be the thought of being restrained by police” (Professional’s interview 2, AMHP).*

These accounts further illustrate how stigma operates through routine practices that misinterpret protective responses as signs of danger. These healthcare barriers are not merely the result of individual bias or interpersonal discrimination; they are embedded in the very architecture of institutions. From healthcare triage systems to social service eligibility criteria, stigma informs how risk is assessed, how resources are allocated, and how urgency is defined.

These accounts further illuminate how stigma operates through what Tyler [26] conceptualises as *stigma power*: the institutional capacity to shape perceptions, responses, and practices in ways that justify coercion, surveillance, or neglect. Here, stigma power is enacted through professional routines that interpret Black men’s protective responses to trauma—such as refusing police entry—not as valid expressions of fear, but as signs of risk, volatility, or non-compliance. This dynamic reinforces a racialised logic in which Black men’s vulnerability becomes obscured by assumptions of threat.

Interpreting stigma through Tyler’s framework [26] shifts the focus from individual prejudice to the systemic reproduction of inequality through organisational processes, statutory mechanisms, and professional cultures. Stigma becomes a structural force—embedded in assessment protocols, police involvement, and risk discourses—that produces and legitimises unequal treatment. In this context, Black men’s expressions of fear or mistrust are not only misunderstood but are reabsorbed into institutional risk narratives that further marginalise them.

Understanding stigma as an embodied and structural form of power makes visible the barriers it creates for racialised communities. It highlights the urgent need for mental health systems to recognise how trauma, racism, and histories of harm shape help‑seeking behaviours—and to respond in ways that prioritise safety, dignity, and cultural attunement rather than defaulting to coercive or risk‑averse interventions.

These accounts underscore the need to interrogate stigma not only interpersonally but as a mechanism of inequality—one that is reproduced through systems, structures, and professional practices. Understanding stigma as a structural force enables a clearer identification—and dismantling—of the barriers it creates, particularly for racialised and marginalised communities.

Importantly, some professionals acknowledged the systemic racism embedded within the very frameworks they operate in. One AMHP stated:

*“Staff feel oppressed and implicitly complicit because the systems they work within are intrinsically racist and the Mental Health Act dominating it is racist. They work day in day out trying to do their best in a system that jars against their values and end up burning out or leaving” (Professional’s interview 3, AMHP).*

This demonstrates that systemic stigma not only marginalises patients but also undermines professionals working toward equity. Understanding stigma as structural enables the identification and dismantling of the barriers it creates for racialised and marginalised communities and for professionals seeking to challenge these inequalities.

This theme highlights the need to interrogate the nuances of professional perceptions, particularly in relation to who is being protected, from what risks, and to whom those risks are directed during detention. These questions are central to understanding how racialised dynamics shape professional decision-making and CAT provision.

Drawing on Critical Race Theory (CRT) [28] and Tyler’s [26] conception of stigma inequality as a socially produced political practice, we recognise that the Black community has been systematically dehumanised, oppressed, and shamed through racial and gendered classification. These processes sustain hierarchies of power and shape how Black men are positioned within mental health systems. Importantly, our findings show that these dynamics extend far beyond what can be resolved through training alone. Professionals at the co‑design event nevertheless emphasised the need for improved learning resources:

*“We need a resource to train all Mental Health practitioners about what we hear today [ImproveAct themes] – Black men's experiences of detention, the police, the inequalities faced and the harm as a result of this.” (Professional, Co‑design event).*

While training remains a necessary component of reform, it is not sufficient when stigma operates structurally. As our earlier findings illustrate, stigma power is enacted through routine institutional practices that misinterpret Black men’s trauma responses as signs of danger, fuelling a racialised risk discourse that obscures vulnerability and reproduces unequal treatment. Understanding stigma as a structural force—intersecting with race, gender, and institutional power—enables us to interrogate the systems that generate these harms and to advocate for change that moves beyond surface‑level professional development. It shifts the focus from individual attitudes to the wider political, organisational, and epistemic conditions that sustain inequality, and clarifies why meaningful transformation requires systemic rather than solely educational interventions.

3 **Race Anxiety and Colour-Blind Racism**

A recurring theme across interviews—particularly with police officers—was a marked anxiety in discussing race. Many participants retreated into colour-blind discourses, insisting that they “*treat everyone the same*” or “*don’t see colour*.” Although presented as commitments to fairness, such statements functioned to deflect responsibility and obscure the racialised realities that shape Black men’s experiences during CAT. This was especially pronounced in moments where professionals were asked to reflect on how race might influence their decision-making or perceptions of risk. Rather than engaging with the question, some participants deflected, minimised, or redirected the conversation. This avoidance reflects what Chantler [32] and McIntyre et al [33] describe as race anxiety; a fear of being perceived as racist or of saying the wrong thing, which leads to silence or defensiveness.

In the quotation below, an explanation for police professionals’ reluctance to talk about race is provided:

*“The police are known as being racist, I think that’s why police officers are reluctant to speak to you, because...no matter what we say we’re perceived that way” (Professionals interview 4, Police participant).*

This defensive stance is echoed in another participant's statement:

*“I’m not racist, I try to treat everyone the same” (Professionals interview 11, Police participant).*

These responses illustrate how colour-blind ideologies and reputation management can obstruct meaningful dialogue about racialised harm. They also reveal how race anxiety operates not only through the marginalisation of racialised individuals but through the institutional silencing of race discourse, which prevents accountability and transformation. These dynamics are central to Critical Race Theory (CRT), which posits that racism is not aberrational but embedded in the ordinary functioning of institutions [28].

The colour-blind approach [46], which purports to treat everyone the same, fails to acknowledge the structural and historical realities of racism and oppression. Such colour-blind approaches are not neutral. They actively erase the lived experiences of racism, reinforce dominant norms, and contribute to epistemic injustice by invalidating Black men’s accounts of racialised distress. While seemingly egalitarian, this perspective fails to acknowledge the power dynamics, oppression, and marginalisation experienced by Black men. It sidelines critical conversations about race and racism, ignoring the broader socio-political context in which anti-Black racism is sustained and maintains racial inequality by denying its existence. Within this discourse, race-based disparities such as the over-detention of Black men are rendered unintelligible, as professionals position themselves as neutral actors within a race-neutral system. The reluctance of police participants to engage in conversations about race, preferring silence over the risk of being perceived as racist [32], reflects race anxiety in how police are responding and could be perceived by Black men as a colour-blind approach. This stance denies the significance of race and racism in shaping lived experiences and professional interactions.

More troublingly, several participants described how biologically essentialist ideas about Black men’s physicality circulated within clinical and policing encounters. As one police officer remarked:

*“Black men are more powerful… if you look at all the athletes...” (Professional’s interview 4, Police participant).*

Such comments echo historic colonial narratives that naturalise Black male bodies as hyper‑physical and threatening [47]. The racialisation of the Black male body in this way is not benign: it actively structures how vulnerability is recognised, how risk is attributed, and how professional responses are justified. Within mental health and policing contexts, these assumptions become embedded in risk assessments, legitimising disproportionate force and reinforcing the idea that Black men are risks being contained rather than individuals in distress.

As one AMHP reflected:

*“We’re told to assess risk, not race. But sometimes I wonder if we’re missing something important by not talking about it.” (AMHP - co‑design event)*

This discomfort reflects the dynamics of race anxiety [32,33], in which professionals fear saying the “wrong” thing, being judged as racist, or appearing unprofessional. Such anxiety frequently results in avoidance, defensiveness, or only superficial engagement with issues of race. In practice, race anxiety reinforces epistemic injustice by sidelining or invalidating the racialised knowledge and lived experiences of Black men, preventing their accounts from being meaningfully integrated into assessment processes. Race anxiety thus becomes more than an interpersonal discomfort; it is an institutional mechanism that polices what can be spoken, forecloses necessary conversations about racism, and sustains the racialised risk discourses that shape CAT.

The persistence of misrecognition in Black men’s emotional expressions, often seen as aggression rather than fear, by some participants, reflects hermeneutical injustice, that is, a collective failure within mental health systems to understand or interpret the experience of racialised trauma, leading to misclassification and over-pathologisation. Professionals reported racial stereotyping, particularly, during police encounters and described how race anxiety shaped their operational roles. This anxiety—rooted in fear of being perceived as racist or of making mistakes—created a climate of heightened caution and increased restriction. This absence of institutional support left many feeling ill-equipped to address racial dynamics, leading to silence, avoidance, and procedural harm.

The concept of ‘race anxiety’ among professionals can be interpreted through CRT as a manifestation of ‘white fragility’ [48] and ‘epistemic dominance’, where whiteness is positioned as the unmarked standard of mental health care. As such, professionals’ discomfort is not an isolated reaction but a systemic avoidance of race-based accountability that maintains institutional status quos.

This theme illustrates how epistemic injustice is embodied – how Black men’s physical presence is interpreted through a racialised lens that delegitimises their knowledge, silences their voices, and justifies coercive responses. It highlights the urgent the need for epistemic openness and anti-racist training in professional practice—an approach that not only acknowledges race anxiety but actively engages with its structural and experiential dimensions.

## Discussion

The findings of this study suggest that Black men are not only overrepresented in CAT but are routinely denied full epistemic recognition: their voices are misinterpreted, their accounts of harm are distrusted, and their cultural idioms of distress are pathologised. The themes collectively highlight the presence of stigma, racism, and epistemic injustice with the CAT of Black men.

The analysis generated three interrelated themes— (1) racial stereotyping and risk assessment, (2) stigma and the denial of care, and (3) race anxiety and colour‑blind racism—which together illuminate how epistemic injustice is produced and sustained within the compulsory assessment and treatment (CAT) of Black men under the Mental Health Act.

Racial stereotyping and risk assessment captures how Black men are professionally stereotyped as inherently risky, dangerous, or unpredictable. These racialised assumptions underpin rapid escalation to coercive interventions, and the systematic undermining of Black men’s credibility during assessment. Accounts of distress, fear, or trauma are frequently reinterpreted as signs of aggression or non‑compliance, reflecting testimonial injustice and fulfilling the objective of examining how racialised framings shape decision‑making within CAT.

Stigma and the denial of care highlights how vulnerability is obscured through structural stigma, influencing triage, risk weighting, and access to care and treatment support. Participants described how Black men’s requests for help were deprioritised or reframed as behavioural problems rather than legitimate clinical needs. These patterns demonstrate how claims to care are differentially valued and reveal hermeneutical injustice in the absence of interpretive resources that acknowledge racial trauma, mistrust, and systemic harm. This theme directly evidences how knowledge about Black men’s experiences is marginalised within clinical pathways.

Race anxiety and colour‑blind racism captures how professionals’ discomfort in engaging with racism—combined with adherence to colour‑blind discourses—reproduces interpretive gaps and prevents epistemically open engagement with Black men’s accounts. This theme shows how professionals may avoid naming racism, minimise its relevance, or reframe it as individual misunderstanding, thereby reinforcing hermeneutical gaps. These findings fulfil the objective of exploring how professionals interpret racial inequality and how understanding, meaning‑making, and credibility are unevenly distributed across CAT detention processes.

Together, these themes demonstrate how epistemic injustice is enacted across assessment, documentation, and decision‑making, revealing the layered mechanisms through which Black men’s knowledge, experiences, and explanatory frameworks are marginalised within CAT.

Our findings thus demonstrate a dual form of epistemic injustice [17] in the CAT of Black men. First, Black men experience testimonial injustice, where their narratives of distress or fear (especially in relation to police encounters) are not afforded equal credibility. Testimonial injustice was most visible during assessment, where credibility deficits shaped how Black men’s accounts of distress, fear, or mistrust are perceived, often resulting in their perspectives being minimised or interpreted through racialised assumptions about risk, aggression, or compliance. It is evident in how professionals, particularly police officers, routinely misread or disbelieve Black men’s accounts of fear, vulnerability, or trauma. Participants described Black men invoking the name of George Floyd, expressing fear of police restraint, or trying to negotiate conditions of safety (e.g., “if the police are not in here, “he’ll be fine”), but these expressions were often interpreted through a racialised lens of risk and non-compliance. The failure to afford credibility to Black men’s own accounts of their situation, especially in the context of their emotional or embodied expressions, is a strong example of Fricker’s [17] credibility deficits. This can lead to inappropriate, excessive, or coercive responses under the guise of clinical decision-making. Serrant’s Silences Framework [34] is particularly useful here because it draws attention to forms of racialised experience that remain unheard within mental health assessment processes to reveal how the voices of Black men are marginalised within these structures. Their narratives of trauma, mistrust, and fear—especially in relation to policing—often become “silenced” by dominant professional discourses of risk, compliance, and behavioural disturbance.

Second, professionals engaged in hermeneutical injustice when they misinterpret expressions of fear, resistance, or trauma as aggression or non-compliance, due to a collective lack of conceptual tools for making sense of racialised emotional expression. During detention elements of hermeneutical injustice become more pronounced, as professionals rely on limited or institutionally dominant interpretive resources that obscure or misrepresent racialised experiences. Similarly, hermeneutical injustice is present in how mental health professionals struggled to understand or contextualise Black men’s distress within broader experiences of racialised harm. The stereotype of being ‘big, Black and dangerous’ not only shaped police responses, but also created interpretive gaps in how fear, resistance, or anger were read as pathological. As one professional noted regarding Black men – as they seem “*very strong, very vocal, and a lot of the time not willing to engage on even simple instructions*:” illustrating a lack of interpretive tools to differentiate between culturally mediated expressions of distress and mental health crisis. These gaps are not just individual but structural, reflecting a profession-wide absence of frameworks that link racial trauma, epistemic exclusion, and mental health presentation.

Addressing epistemic injustice requires more than cultural competence or individual-level awareness [49]. It demands a transformation in institutional listening, co-production, and epistemic openness, particularly through recognising Black men as legitimate knowers of their own mental health realities.

Applying the lens of epistemic injustice allows us to interpret these findings not merely as instances of interpersonal bias or unconscious racism, but as part of a wider epistemic system in which Black men are systematically denied recognition as reliable narrators of their own experiences. This reinforces a racialised mental health system in which knowledge generated by professionals is seen as legitimate, while knowledge expressed by detained individuals, particularly those racialised as Black, is either discounted or reframed through dominant (White, Eurocentric, clinical) paradigms.

Actioning epistemic injustice requires institutional as well as interpersonal change. Mental health professionals must be trained not only in cultural humility but also in epistemic humility, recognising the limits of their interpretive frameworks and creating space for co-produced, lived experience-led knowledge. Recognising epistemic injustice within mental health detention processes necessitates a fundamental rethinking of how mental health professionals engage with knowledge, power, and racialised experience. This includes ensuring that the Mental Health Act 2025 Code of Practice includes provisions for narrative validation, inclusive advocacy, alternative epistemologies of wellbeing and shared decision-making structures that challenge credibility hierarchies [50]. In this way, the routine silencing and distortion of Black men’s experiences under the guise of clinical neutrality can begin to be redressed.

These injustices are not merely interpersonal but reflect structural failures in the training, policy, and frameworks that govern mental health practice. Without addressing epistemic injustice, racial disparities in CAT will persist, not just in who is detained, but in how their voices are received, understood, and acted upon. Training must support professionals not only to hear but to understand *expressions* of distress in racialised contexts.

This discussion forms a clear bridge to the following Practice, Organisational, and Policy recommendations which build on our findings to outline the shifts in professional practice, organisational culture, and legislative reform required to embed epistemic justice within mental health CAT systems.

## Recommendations

### Challenging epistemic injustice in CAT: Practice, Organisational, and Policy Actions

1 **Immediate Implications for Professional Practice**

Professionals involved in CAT (including police, AMHPs and social workers, psychiatrists, psychologists, and nurses) should be supported to develop epistemically reflexive practice. This entails cultivating awareness of how dominant racialised assumptions shape the attribution of credibility and legitimacy in clinical and practice encounters. Crucially, professionals must learn to interrogate their responses to resistance or fear, in Black men’s presentations—not as inherent indicators of risk, but as potentially rational responses to historic and ongoing racialised harm.

We therefore propose a shift from cultural humility to epistemic openness—the recognition that one’s interpretive frameworks are partial and may exclude or misread Black men’s narratives. Embedding this shift in professionals’ training requires:

(i) Lived‑experience–led training that centres Black men’s narratives and explicitly teaches how testimonial and hermeneutical injustice manifest in CAT.
(ii) Counter‑narratives and participatory storytelling integrated into workforce development (aligned with the principles of the Silences Framework) to expand available interpretive resources.
(iii) Reflexivity tools for risk assessment, supporting professionals to identify when and why Black men’s accounts may be dismissed or downplayed—and how to correct for this in situ.
**2. Organisational Conditions for Epistemic Openness**

Beyond individual practice, mental health institutions must be epistemically open—creating genuine space for alternative knowledge systems, peer‑led expertise, and community‑based epistemologies to inform care planning and policy. While training can initiate this orientation, sustained change requires structural and relational commitments, including:

(i) Structured reflexive dialogue within multidisciplinary forums to surface tacit assumptions and challenge racialised interpretive habits.
(ii) Revised documentation practices that legitimise and record service‑users’ explanatory frameworks alongside clinical formulations.
(iii) Strengthened supervision that encourages critical examination of racialised reasoning and its effects on credibility judgments.
(iv) Lived‑experience authority embedded in governance so that experiential knowledge meaningfully shapes decisions, pathways, and service design.
**3. Governance, Accountability, and Audit**

To translate epistemic openness into accountability:

(i) Formally recognise epistemic harm within complaints, safeguarding, and governance processes.
(ii) Co‑produce training and policy guidance with Black communities, including mechanisms for service users and families to document how their accounts were received or ignored.
(iii) Reform written narratives and referrals to minimise bias—reducing “warning language” and racially coded descriptors that distort CAT pathways.
(iv) Introduce epistemic impact assessments in audits, asking not only whether outcomes were equitable, but whether people’s accounts were believed, understood, and acted upon.
**4. Alignment with UK Policy Frameworks**

These recommendations align with national efforts such as the Patient and Carer Race Equality Framework (PCREF) [51] and the work of the NHS Race & Health Observatory. The study’s findings can help operationalise PCREF by specifying how epistemic justice is enacted in everyday practice. At the same time, we note current limitations with PCREF: uneven implementation, lack of statutory enforcement, and the absence of mandated racial‑trauma‑informed assessment tools. Our proposals [40] address these gaps by recommending binding safeguards—for example, minimum standards for trauma‑informed assessments and formal rights to culturally contextualised understanding—so epistemic justice becomes a deliverable requirement, not a discretionary aspiration.

5 **Legislative and Policy Reform**

Reform of legislation governing mental health detention and care should explicitly embed epistemic justice as a guiding principle in the Mental Health Act 2025 Code of Practice. Individuals subject to compulsory detention should have a formal right to be understood within the context of their racialised and cultural experience. We therefore recommend:

i Stronger safeguards for voice, advocacy, and co‑decision‑making, and
ii A revised Code of Practice (co‑developed with the workforce and people with lived experience) that provides actionable guidance for supporting individuals to receive person‑centred, beneficial care and treatment, explicitly incorporating epistemic justice as a guiding principle. Thus, operationalising epistemic justice as a legal and clinical imperative rather than a discretionary goal.

Articulating these recommendations underscores the practical implications of adopting an epistemically open stance within compulsory detention settings, clarifying how injustice manifests at each stage of detention and identifies concrete strategies to disrupt these dynamics, thereby strengthening both the analytical contribution and the applied relevance of the study. Without such measures, the reformation agenda risks reproducing existing power asymmetries under new language.

#### Strengths and limitations.

This study makes an original contribution to scholarship on racism and compulsory detention by integrating epistemic injustice, stigma inequality, critical race theory, and the Silences Framework within an Experience‑Based Co‑Design (EBCD) framework, while examining compulsory detention from the perspective of professionals rather than service users. This theoretical–methodological synthesis advances existing work in three key-ways.

First, the study offers a novel epistemic framing for understanding how racialised knowledge hierarchies and testimonial deficits shape compulsory assessment and treatment. Whereas previous research has focused on structural racism within detention practices, this study offers an original contribution to the field by explicitly theorising how professionals’ interpretive practices contribute to everyday forms of epistemic harm. In doing so, it illuminates how racialised assumptions, stigma inequality [26] and credibility deficits [17] are embedded in professional’s reasoning and risk assessment.

Second, the study provides an innovative methodological contribution through its use of EBCD to analyse professional reasoning in a racialised context. Although EBCD is well established as an approach for centering service‑user experience, its application to exploring professionals’ accounts—particularly in settings characterised by coercion and racial inequality—is rare. The study demonstrates how EBCD can generate nuanced insights into how professionals make sense of racialised encounters, navigate institutional pressures, and either reproduce or disrupt racial injustice.

Third, by focusing on professionals’ experiences, the study adds a distinctive perspective to existing research, which has centered on service‑user experiences of racism in mental health systems. This professional lens reveals how practitioners narrate, justify, and negotiate racialised practices within CAT, offering granular insights into the mechanisms through which racism is enacted, normalised, or challenged at the point of assessment. In doing so, the study advances rather than duplicates existing literature, contributing a new layer of understanding to the scholarship on racialised mental health inequities.

By positioning this contribution in relation to established literature on racism and compulsory detention—including the work of Edge [5], Keating [6], and Nazroo et al [9],—the study demonstrates how the innovation lies in the integration of epistemic framing, EBCD methodology, and a focus on professional perspectives, thereby advancing rather than reiterating existing scholarship.

A limitation of the study was the inability to recruit paramedics and mental health nurses. Feedback from gatekeepers indicated that this was partly due to the sensitive nature of the research topic. During the post-COVID recovery period there were significant demands on staff, and staff shortages. Gatekeepers suggested that the limited participation of nurses and paramedics may have been influenced by the sensitive nature of the research topic, as well as their comparatively peripheral role in formal mental health assessments, relative to other professionals such as AMHPs and psychiatrists, who were recruited.

## Conclusion

This study shows that the CAT of Black men under the Mental Health Act is shaped by interconnected dynamics of racial stereotyping and risk inflation, structural stigma that delays or denies care, and race anxiety and colour‑blind racism that inhibit open engagement with racial inequality. Across professional groups, Black men were routinely framed as inherently risky, and their expressions of fear, distress, and trauma were frequently reinterpreted through tropes of dangerousness rather than understood as indicators of vulnerability. These patterns produced testimonial injustice, whereby Black men’s accounts were disbelieved, minimised, or reframed through dominant institutional narratives.

Stigma operated structurally through triage and risk‑weighting processes that consistently prioritised “risk to others” over “risk to self,” limiting opportunities for preventative and therapeutic support. Race anxiety and colour‑blind discourses further contributed to hermeneutical injustice, as professionals lacked conceptual and organisational resources to recognise and interpret racialised expressions of distress. Trauma was therefore frequently misattributed to non‑compliance, resistance, or aggression. 

Collectively, our findings demonstrate that epistemic injustice is systemically embedded in the routine functioning of CAT rather than confined to isolated instances of bias. Addressing this requires more than generic equality training. It demands the embedding of epistemic humility, racial and trauma‑informed assessment, and co‑produced, lived‑experience‑led knowledge across the full spectrum of practice, policy, and governance. Future reform of the Mental Health Act Code of Practice, along with related national frameworks, must center equality principles to ensure that services do not continue to reproduce epistemic harm.

The future of Black men’s mental health depends on professionals acting in ways that recognise the person in their full social and historical context, acknowledge the traumatic effects of racism, and affirm Black men as credible knowers of their own experiences. Ultimately, achieving justice in compulsory mental health care requires a system in which Black men’s accounts are heard, understood, and acted upon as a fundamental obligation of ethical, clinical, and societal responsibility. Ultimately, meaningful change will depend on recognising Black men not as objects of risk, but as bearers of knowledge whose accounts must be heard, understood, and acted upon as a matter of both clinical responsibility and social justice.

Our findings demonstrate epistemic injustice in the CAT of Black men. These injustices must be understood, challenged, and reflected upon as part of mental health professionals’ clinical and professional practice. Working in a climate of fear and anxiety has significant consequences for the quality and fairness of CAT involving Black men. For meaningful change, organisations and professionals must recognise how their own race anxieties, interpretive habits, and organisational constraints shape Black men’s experiences of detention.
